# Mental files theory of mind: When do children consider agents acquainted with different object identities?

**DOI:** 10.1016/j.cognition.2017.10.011

**Published:** 2017-11-16

**Authors:** Michael Huemer, Josef Perner, Brian Leahy

**Affiliations:** a Centre for Cognitive Neuroscience, University of Salzburg, Hellbrunnerstraße 34, 5020 Salzburg, Austria; b Department of Psychology, University of Salzburg, Hellbrunnerstraße 34, 5020 Salzburg, Austria; c Department of Linguistics, University of Konstanz, Universitätsstraße 10, 78464 Konstanz, Germany

**Keywords:** Mental files, Theory of mind, False belief, Intensionality, Acquaintance

## Abstract

Mental files theory explains why children pass many perspective taking tasks like the false belief test around age 4 (Perner & Leahy, 2016). It also explains why older children struggle to understand that beliefs about an object depend on how one is acquainted with it (intensionality or aspectuality). If Heinz looks at an object that is both a die and an eraser, but cannot tell by looking that it is an eraser, he will not reach for it if he needs an eraser. Four- to 6-year olds find this difficult (Apperly & Robinson, 1998). We tested 129 35- to 86-month olds with a modified version of Apperly and Robinson’s task. Each child faced four tasks resulting from two experimental factors, *timing* and *mode of information*. Timing: Children saw Heinz learn the die’s location either *before* or *after* they learn that the die is an eraser. Mode of information: Heinz learns where the die is either perceptually or verbally. When Heinz’ learning is verbal, he never perceives the die at all. We found that Apperly and Robinson’s problem occurs only in the *seen-after* condition, where Heinz *sees* the die *after* children had learnt that it was also an eraser. It vanishes when Heinz learns where the die is *before* children learn that it is also an eraser. The problem also vanishes when Heinz learns where the die is purely verbally (e.g., “*The die* is in the red box”) and never sees it. This evidence lets us refine existing mental files theory, and eliminate several alternatives from the literature.

## 1 Introduction

Labels influence the ways we think about objects. This is particularly salient for objects with dual functions. For instance, a rubber die that can be used to erase pencil marks is both a *die* and an *eraser*. So it can be thought of (or spoken of) as a die or as an eraser. Three year old children have problems with multiple identities like this. This explains children’s reluctance to use different names for the same object (Flavell, 1988; Markman, 1989; Markman & Wachtel, 1988: mutual exclusivity bias) and their problems making the appearance-reality distinction (Flavell, Flavell, & Green, 1983). Dual identities give rise to identity statements like “The die is the eraser”. This statement is informative when one has learned about the die and eraser separately, but does not know they are the same object. Three year olds have problems making sense of such statements. Psycholinguists (Clark, 1997; Tomasello, 1999) note that calling an object by a different name (label) puts a different perspective on the object. So it is interesting that children’s problems with identity statements relate to their problems understanding another person’s perspective in the traditional false belief task (Perner, Mauer, & Hildenbrand, 2011). When one object can be thought of in multiple ways, we will say that there are multiple possible *conceptual perspectives* on that object.

Understanding that others may think of an object under one conceptual perspective but not another is even more difficult. Russell (1987) first explored this issue. In his stories George realizes that his watch has been stolen. Children learned that the thief had curly red hair and were asked, “Can we say that George was thinking: ‘I must find the man with the curly red hair who stole my watch’?”. Children up to 6 or 8 years find such judgements difficult (Kamawar & Olson, 1999, 2009, 2011). Apperly and Robinson (1998, 2001, 2003) avoided asking for explicit metalinguistic judgments in their *Heinz scenario*. Four- to 6-year-old children were shown a rubber die, which they identified as a die. They were then shown that the die was also an eraser. Puppet Heinz entered the scene and observed with the child that the die/eraser was put into one box and a standard eraser into another box. At this age most children pass the false belief test; when asked, “Does Heinz know that the die is an eraser?” they mostly answered correctly with “no”. Yet when asked, “Where will Heinz go to get an eraser?” they indicated the location of the die/eraser as often as the location of the standard eraser: A curiously incoherent pattern of responses.

Sprung, Perner, and Mitchell (2007) and Perner, Huemer, and Leahy (2015) found that this pattern occurs in a developmental window between passing first-order false belief tasks and passing second-order belief tasks about 2 years later. We refer to these children as “(+ −)” because they pass the first-order test but fail the second-order test. This was tested as follows: Max puts his chocolate into box 1 and leaves. The chocolate is moved to box 2. Max returns; children face two test questions: (1) “What will Max say if we ask where his chocolate is?” (2) “What will Max say if we ask whether he knows where his chocolate is?”. Correctly answering (1) requires that participants understand where Max *thinks* his chocolate is; a first-order problem. Correctly answering (2) requires that participants understand that Max *thinks* that he *knows* where the chocolate is; a second-order problem.

**Fig. 1:**
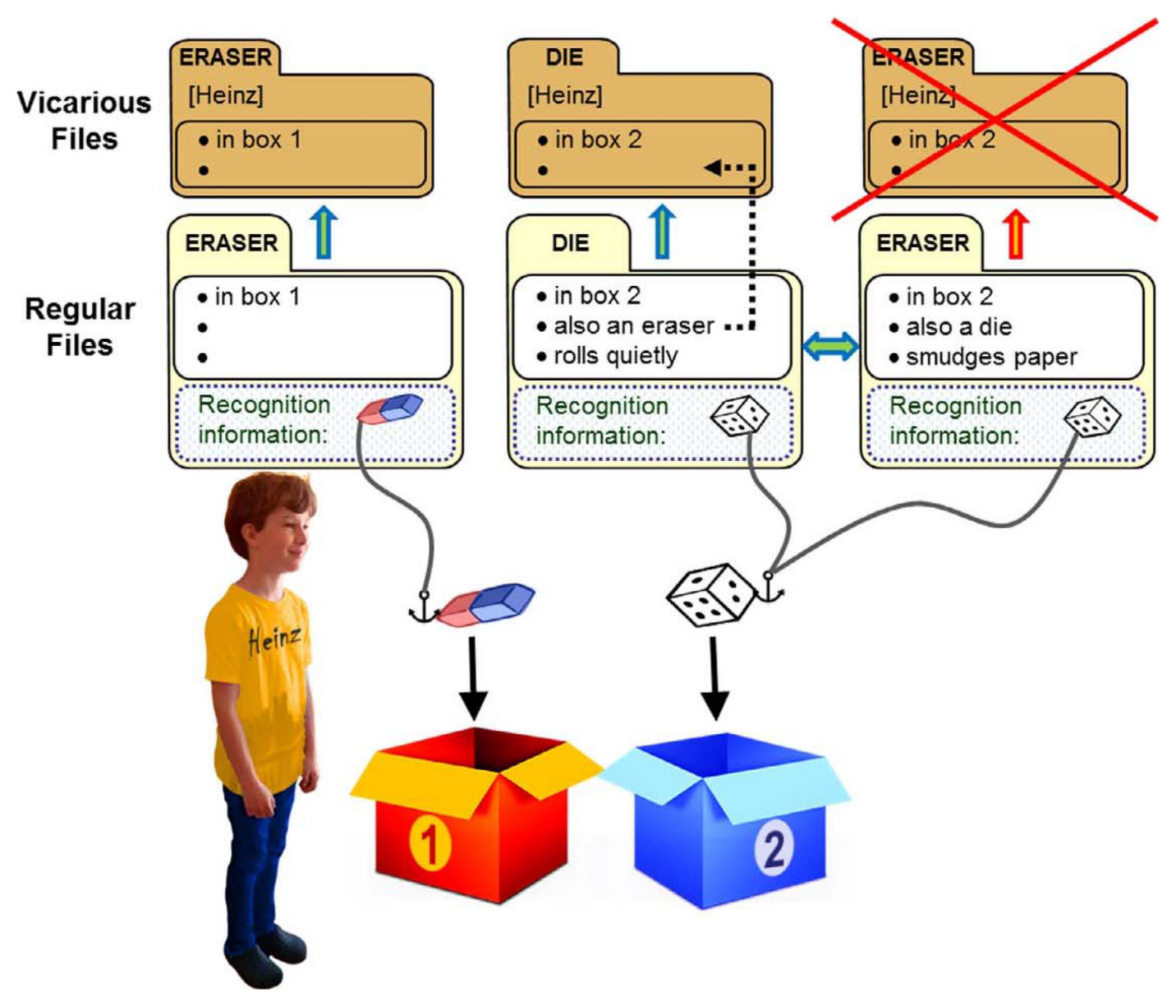
Mental files analysis of Apperly and Robinson’s (1998) Heinz scenario for (+ −) children. The child, seeing the scenario, has a regular file for the standard eraser and two regular files for the die/eraser. When the child sees Heinz seeing the objects, she deploys a vicarious file indexed to Heinz for each of her own regular files. However, the vicarious eraser-file for the die/eraser is an error, since Heinz cannot tell by seeing that the object is an eraser. The erroneous file is therefore crossed out in the figure. The child has registered the information “also an eraser” on her regular die-file, and correctly does not transfer this information to the vicarious die-file (that the child correctly does not execute this transfer is indicated by the broken arrow). The anchors indicate the object a file is tracking.

Mental files theory (Perner et al., 2015) explains the curious pattern of responses in the Heinz scenario by distinguishing that an object is conceived under a particular concept from the information one has about the object. A dual object like the die/eraser can be conceived as a *die* (represented in the mind by a file headed “die”) and as an *eraser* (represented by a file headed “eraser”). These files, which store the participant’s information about objects in the environment, are called *regular* fi*les*. The regular die-file records the object’s qualities as a die, e.g., that it is in box 2 and that it rolls quietly, as well as perceptual information that helps the child recognize the object at later times. The regular eraser-file records its qualities as an eraser, e.g., that it is in box 2 and that it erases poorly (smudges the paper), again along with recognitional information. Fig. 1 illustrates this for the Heinz scenario (including a regular file for the standard eraser). Each file holds information that enables the participant to recognize and re-identify the object it tracks. The two files that track the same object (die/eraser) in Fig. 1 are linked by a double arrow, which captures the participant’s understanding that the die is the eraser. Because the files are linked, the participant has access to all information contained in one file (e.g., the die-file) even when thinking about the object from the other conceptual perspective (e.g., as an eraser).

According to mental files theory, agents use another kind of file, *vicarious files*, to model another agent’s perspective on the world. Vicarious files are indexed to the agent whose beliefs they track. Much as regular files store information about objects the child is tracking, vicarious files store information about the objects that the child sees other agents tracking. So as the child’s perspective is reflected in her regular files, the agent’s perspective is reflected in her vicarious files.

When a child sees an agent track an object, she must *vertically link* the vicarious file indexed to the agent to her regular file for the same object, thereby marking that the two files track the same object. Vertical links, unlike links between regular files, allow only restricted flow of information. In the Heinz scenario there will be a vicarious eraser-file indexed to Heinz and vertically linked to the regular file for the standard eraser (Fig. 1, upper left). There will also be a die-file indexed to Heinz and linked to the regular die-file for the die/eraser. The file contains Heinz’ information about the dual object when conceived of as a die, e.g., that it is in box 2.

Mental files theory proposes that (+ −) children make systematic errors in managing this filing system. These errors explain why (+ −) children say that Heinz does not know that the die is an eraser, but act as though he might reach for the die when he needs an eraser. We know that children develop an understanding of knowledge formation through visual perception between 3 and 4 years of age (Hogrefe, Wimmer, & Perner, 1986; Sodian, Thoermer, & Dietrich, 2006). So (+ −) children recognize that Heinz cannot learn that the die is also an eraser by just looking at it. Hence, they correctly leave out the information “is also an eraser” on the vicarious die-file and answer the question, “Does Heinz know that the die is an eraser?” correctly with “no”.

However, (+ −) children create too many vicarious files (see Fig. 1, where a vicarious eraser-file is crossed out to mark its illegitimacy). This gratuitous vicarious file misleads children: When asked where Heinz will look for an eraser, they check the vicarious files for Heinz, find two files headed “eraser”, and choose one of them at random. Hence their curiously incoherent behavior.

Where does this gratuitous vicarious file come from? Perner and Leahy (2016) claimed that (+ −) children copy their regular files to vicarious files, but sometimes make copies when they shouldn’t. Since they have two files for the die/eraser, Heinz, too, gets the two by copying.

This proposal of copying regular files and their labels has problems. If vicarious files were always copies of regular files with labels, then there could never be a vicarious file that does not have the same label as a corresponding regular file. This is implausible because Perner et al. (2015, Fig. 7) report that (+ −) children can assign a vicarious file for which they have no corresponding regular file with the same label. So Perner and Leahy’s copying proposal needs amendment. In this paper we will speak of “deploying vicarious files” instead of “copying regular files”. (+ −) children systematically deploy too many vicarious files.

We now discuss how (+ −) children deploy vicarious files. We discuss two cases. First we describe how vicarious files are deployed when the agent’s contact with the object is only perceptual (and not verbal). Then we describe how they are deployed when the agent’s contact is only verbal (and not perceptual).

If an object appears in an agent’s perceptual space without any linguistic description, one or more vicarious files are deployed for that object, indexed to the agent. In the case of the die/eraser there will be two files, one for the object as a die and another for it as an eraser. After all, not only a die but also an eraser is within Heinz’s visual field. ^2^ This excess deployment accounts for (+ −) children’s characteristic error.

To avoid this excess deployment, one has to understand that Heinz cannot think of the object as an eraser unless he can tell from just looking at it that it is an eraser. To use this knowledge to constrain deployment of vicarious files, children must concern themselves with the question of which conceptual perspectives are available from Heinz’s perspective. This is a concern about second-order perspectives. That is why children master it only when they are able to handle second-order problems like the second-order false belief task.

Our second case describes vicarious deployment when the agent’s contact with the object is purely verbal. Following DRT (Kamp & Reyle, 1993) and file change semantics (Heim, 2002), a referring expression like “a die” causes deployment of a new file, unless an existing file already responds to that expression. The new file is given the label used in the expression and no other file is deployed. So in our case with Heinz, children check whether an existing vicarious file indexed to Heinz responds to “a die”. Since there is none, a new vicarious file labelled “die” is deployed. No other file is deployed.

A testable implication follows: (+ −) children should not commit the Apperly and Robinson error when Heinz’ information is purely verbal: If Heinz does not get to look at the die but is only told that there is *a die* in box 2.

Vicarious file deployment also suggests that deployment is time sensitive. Files are deployed only *at the time* when Heinz encounters the object in his visual field or when he hears the referential expression “a die”. This has a testable implication: The curiously incoherent pattern should only appear when participants already know the die is an eraser when Heinz encounters the object. If (+ −) children only know it as a die when Heinz encounters it, they will only deploy a die-file, since they don’t know it is also an eraser. When children learn about the die’s dual nature only later, after Heinz’ encounter with the die, they will not deploy an excess vicarious eraser-file. Since excess files cause the incoherent pattern, incoherence should disappear.

**Fig. 2:**
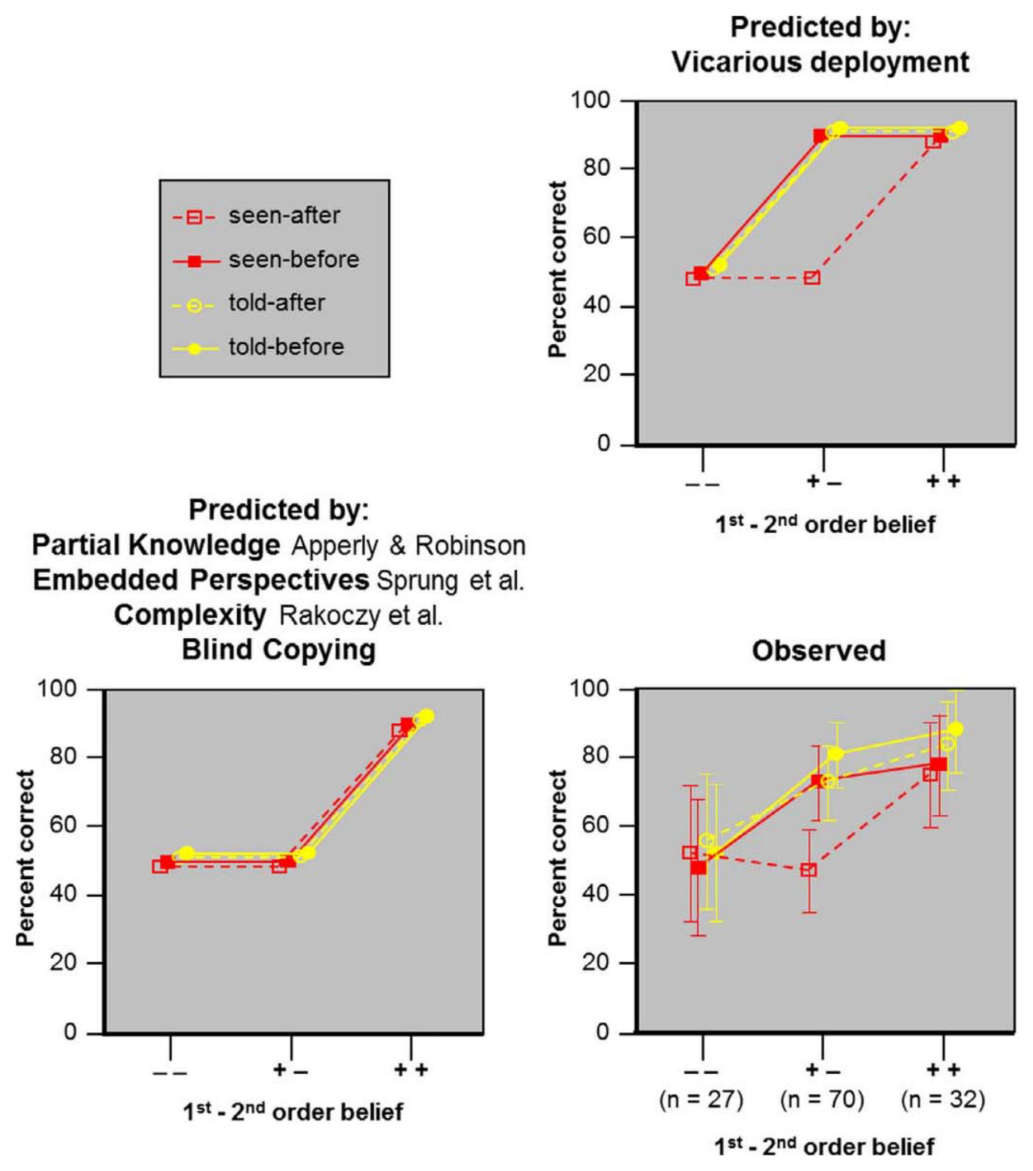
Predicted and observed percentage of correct responses to the where-look question. The x-axis in each panel shows the three categories of children according to their failing or passing the 1st and 2nd order false belief test (pass criterion: At least one of 2 questions correct). The four lines correspond to the four conditions. For better visibility and to avoid overlap, theoretical predictions of 100% correct are scattered around 90%. Error bars for the observed data show ± CI. Note: The prediction by Apperly and Robinson (1998, 2003), also applies to the alternative explanations by Rakoczy et al. (2015) and Sprung et al. (2007) (see below and discussion section for details).

We tested these implications by manipulating the timing and modality of Heinz’ information. In our experiment Heinz receives information about the object either *before* or *after* children learn of its dual nature (timing). Heinz gets to know of the object either by *seeing* it or by being *told* about it (modality). This yields four conditions: seen-before, seen-after, told-before, and told-after. The predictions based on vicarious file deployment for (+ −) children are graphically depicted in the upper right panel of Fig. 2. Only the original condition used by Apperly and Robinson (seen-after) should be difficult. This difficulty should vanish from the three other conditions, either because the information is only verbal (told-before and -after) or because children do not yet know that the die is also an eraser when Heinz first encounters this object (seen-before).

Our experimental design also allows us to test several existing theories that all make the same predictions (see Fig. 2). Apperly and Robinson (1998) explained their finding as follows: In order to figure out where Heinz will look for an eraser, children ask of each box, “Does Heinz know there is an eraser in this box?” This question has two readings: The opaque reading, “Does Heinz know that there is an object in this box that he knows is an eraser?” and a transparent reading, “Does Heinz know that there is an object in this box, which is actually an eraser (whether or not he knows it’s an eraser)?”. Children do not represent Heinz’ knowledge as partial, and so they cannot find the opaque reading. ^3^ They are left with the transparent reading. Since Heinz has seen the die in the box, and the die actually is an eraser, the answer to the transparent reading is “yes” (p. 297). This seduces some children to indicate the location of the die-eraser when asked where Heinz will go to get an eraser. Heinz’ knowledge is partial in all four conditions of our study. Consequently, this theory predicts no change in difficulty across conditions.

Rakoczy, Bergfeld, Schwarz, and Fizke (2015) proposed that children’s problems are due to the challenges of reference resolution posed by ambiguity. ^4^ In the Apperly and Robinson scenario there are two erasers, and then the children are told that Heinz wants “an eraser”. The child must resolve the ambiguity of this expression: “An eraser” plausibly refers to either eraser, but the child must calculate that since Heinz only knows that the standard eraser is an eraser, “an eraser” must refer to the standard eraser. Perhaps children fail to solve this reference resolution problem correctly. All four conditions of our study involve ambiguous reference and so pose similar reference resolution problems. So if children’s difficulty with Apperly and Robinson’s task arises from a struggle with reference resolution, all four conditions of our study should be equally difficult.

Sprung et al. (2007) attributed children’s difficulty to embedding of perspectives created by having to decide whether the eraser perspective on the object is available from Heinz’s limited perspective. Since this is a second-order perspective taking problem, Sprung et al. predicted that children should not be able to master it before they are able to solve second-order belief attribution ((+ +) children). However, there is nothing in this proposal that would suggest that any one of our three new conditions would lessen this second-order problem.

We see that these three explanations of the original Apperly and Robinson data all make the same prediction that our new conditions should make no difference. This predicted pattern is shown in left bottom panel of Fig. 2.

## 2 Method

### 2.1 Participants

One-hundred twenty-nine children (78 female, 51 male) from five kindergartens and two elementary schools (ages 35–86 months; median age 66 months; S.D. = 11.8) participated in this experiment. Parents were previously informed and gave written consent to their child’s participation.

### 2.2 Design

Each child faced four dual description ‘intensionality’ tasks as a result of two experimental factors. *Timing*: Children see Heinz learn where the die/eraser is either *before* or *after* they learn about the dual nature of this object. *Mode of information*: Heinz learns where the die/eraser is either *perceptually* or *linguistically*. This yields four conditions: seen-after (traditional version), seen-before, told-before, and told-after. We used different materials in each condition (pen/lamp, ball/rattle, dolphin/squirt gun, die/eraser) in a counterbalanced fashion (see below). We used the vocabulary sub-test of the HAWIK IV (Petermann & Petermann, 2010) to assess verbal intelligence, and two false belief tasks, each with a first-order a second-order belief question.

The order of the seven tasks was: Verbal intelligence test, a *seen*- and a *told*-task (counterbalanced order), a false belief task, *seen*- and *told*-task (counterbalanced order), and another false belief task. Each condition occurred first equally often. The task in second position alternated for *seen* and *told*, and for *before* and *after*, e.g.: *seen*-*before*, *told*-*after*. The following tasks repeated the sequence but with *before* and *after* exchanged, e.g.: *seen*-*after*, *told*-*before*. The resulting four orders of conditions were counterbalanced with a Latin Square design of story material (pen/lamp, ball/rattle, dolphin/squirter, or die/eraser) resulting in 16 combinations. Each combination was given with one of the false belief tasks with either material (marble, book) in positions 3 and 6, respectively, resulting in 32 different sequences altogether. Each child was assigned randomly to one of the sequences until each sequence had been used once whereupon a new random assignment was begun until all children had been tested.

### 2.3 Procedure

Children were tested in 15 min sessions by a male experimenter in a quiet room of their kindergarten or elementary school.

#### 2.3.1 Dual description ‘intensionality’ tasks

**Fig. 3:**
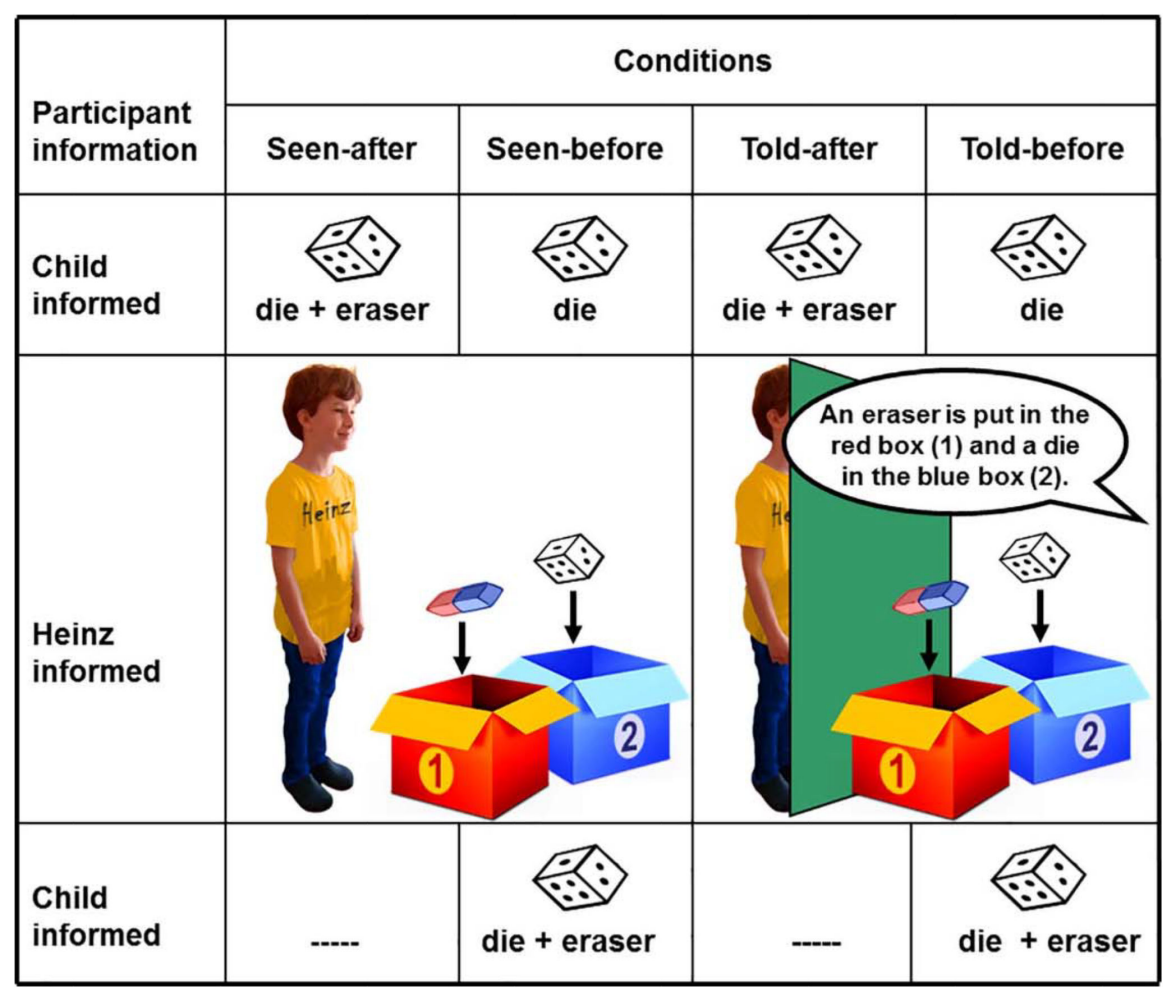
Illustration of experimental conditions with factors *timing* and *mode of information*. *Timing*: Children see Heinz learn where the die/eraser is either *before* or *after* they learn about the dual nature of this object. *Mode of information*: Heinz learns where the die/eraser is either *perceptually (seen)* or *linguistically (told)*.

Our procedure (see Fig. 3) had four phases: Familiarization, Participant Information, Agent-Object Contact, and Test Questions. All conditions began with Familiarization and ended with Test Questions. In *before* conditions, Agent-Object Contact came *before* Participant Information. In *after* conditions, Agent-Object Contact came *after* Participant Information.

*Familiarization.* Puppet Heinz was introduced to the child and then put away. The experimenter showed the child empty red and blue boxes.

*Participant Information.* After pointing out that Heinz is playing elsewhere and cannot see or hear what is going on, the experimenter introduced the die/eraser and demonstrated that the die is also an eraser. The experimenter repeated that Heinz could not see the demonstration and asked:

*Know-question 1.* “Does Heinz know that the die is also an eraser?”

*Agent-Object Contact.* In the *seen conditions* Heinz entered and was shown a standard eraser with the words, “Here we have an eraser” and the die/eraser with, “Here we have a die”. Heinz and the child were told to watch carefully while the experimenter put the die/eraser in one box and the standard eraser in the other. In the *told conditions*, the child was informed that Heinz will sit behind a screen where he can hear us but cannot see what we do. With Heinz behind the screen the experimenter put the objects in boxes while saying: “Heinz, listen carefully. Here we have a die and an eraser. I am putting the eraser in the red box. I am putting the die in the blue box”.

*Test Questions.* Three test questions followed in this order:
*Memory question:* “Where had we put the die in the beginning?”
*Know-question 2:* “Does Heinz know that the die is also an eraser?”
*Where-look* question: “Heinz wants to have an eraser - where will Heinz look for an eraser?”

#### 2.3.2 False belief tasks

Two unexpected transfer false belief tasks with a first-order and a second-order test question (modeled after Perner & Howes, 1992; Sprung et al., 2007) were presented. Max put an object (without a dual aspect, e.g. a marble) into box 1 and went away. The experimenter moved the object from box 1 to box 2. Max returned and the child answered questions in this order:

*Memory* question: “Where did Max put the marble in the beginning?”
*Reality* question: “Where is the marble now?”
*Test* question *1st order*: “If we ask Max: ‘Where is the marble?’ What will he say?”
*Test* question *2nd order*: “If we ask Max: ‘Do you know where the marble is?’ What will he say – will he say ‘Yes, I know’ or ‘No, I don’t know’?”

## 3 Results

### 3.1 False belief tasks

Children performed well on the *memory* and *reality* questions in the false belief tasks; overall they answered 96% of these questions correctly. The consistency in performance of the children over both false belief tasks was high for the first-order belief question as well as the second-order belief question (Φs = 0.88 and 0.65, respectively). Twenty-seven children answered both first-order belief questions incorrectly and were classified (− −) (mean age = 51, S.D. = 11.3). Seventy children passed at least one of the first-order questions, but failed on both second-order belief questions and were classified (+ −) (mean age = 67, S.D. = 8.2). The remaining 32 children were classified (+ +) (mean age = 74, S.D. = 8.3).

### 3.2 Dual description aspectuality tasks

Ninety-six percent of *memory* questions and 89% of *know* questions were answered correctly.^5^ Fig. 2 (bottom right panel) encodes percentages of correct answers to the critical where-look question along with predictions from the three theories in the literature as well as the *vicarious deployment* theory. Predictions for each category of children are as follows.

All accounts predict that (− −) children will answer the where-look question by choosing randomly between the locations of the die-eraser and the standard eraser. Children who do not pass the first-order false belief task can’t use vicarious files effectively, either because they can’t deploy vicarious files (Perner & Leahy, 2016; Perner et al., 2015) or because they can’t link them (Perner, 2016). So they use their regular files to answer the where-look question. Because there are regular eraser-files for both the standard eraser and the die/eraser in all four conditions, children can choose either location. For the four conditions answers were correct between 48% and 56% (none different from chance, Binomial tests: all p ≥ 0.701), and there was no reliable difference between the conditions (Wilcoxon signed rank test: all p ≥ 0.564).

The behavior of (+ −) children teases the theories apart. As described in the introduction, all existing theories predict that all conditions will be difficult. On the vicarious deployment theory only the seen-after condition should be difficult.

Observed data match the predictions of the vicarious deployment theory (Fig. 2 top right panel). In the seen-before and both told conditions (+ −) children gave 73% or more correct answers (each above chance, Binomial test: all p ≤ 0.001). There was no reliable difference between the conditions (Wilcoxon signed rank test: all p ≥ 0.157). In the seen-after condition children gave only 47% correct answers, which is not different from chance (Binomial test: p = 0.720) but is significantly lower than the performance in the other conditions (Wilcoxon signed rank test: all p ≤ 0.002).

For (+ +) children, all theoretical accounts predict that all four conditions will be easy. This was observed: In all conditions children’s performance was at 75% or more correct (each above chance, Binomial test: all p ≤ 0.007), with no reliable difference between conditions (Wilcoxon signed rank test: all p ≥ 0.157). Additionally, mental files theory links the improvement in the seen-after condition from (+ −) children to (+ +) children to the mastery of second-order problems. This improvement is significant even when accounting for age and verbal competence (logistic regression: Wald (1) = 4.01, p = 0.045).

## 4 Discussion

Our data replicate Apperly and Robinson’s (1998, 2001) finding: (+ −) children struggle with the where-look question in the seen-after condition, despite asserting that Heinz doesn’t know that the die is an eraser. In our three novel conditions, they performed about as well as (+ +) children. This is in line with the proposed *vicarious deployment* version of mental files theory, summarized as follows for our three types of children:

(− −) *children use their own regular files to predict others’ actions.*
(+ −) *children use vicarious files to predict others’ actions. They assign vicarious files for an object when the agent gets information about the object. If the information is purely linguistic, children check for an existing vicarious file with an appropriate label. If there is none, a new vicarious file with the appropriate label is deployed. No other file is deployed. If the information is purely perceptual, children deploy files that match all their own conceptions of the object. This can lead to excess vicarious files. These children can register lack of knowledge about the object’s properties by leaving the information off the vicarious file. But they cannot yet use this information to restrict deployment of excess files.* (+ +) *children also use vicarious files to predict others’ actions, but they are more selective. They only admit vicarious files that capture conceptual perspectives available to the agent, i.e., they admit only files with labels whose corresponding property the agent can detect. Thus they avoid the excess deployment that* (+ −) *children succumb to.*

Applied to our four conditions this means that (+ −) children do not deploy excess vicarious files in the information-before conditions, since the agent shares all the child’s conceptions when the information is given. In the told-after condition there are no excess files since the verbal statement containing “a die” only gives rise to a die-file for the object.

This contrasts with the seen-after condition. Heinz sees an object that the child knows is both a die and an eraser. In order to assign the right vicarious files, the child needs to recognize which conceptions are available from Heinz’ perspective. This second-order question is beyond the abilities of (+ −) children, who comprehend first-order but not second-order mental states.

The vicarious deployment theory is sensitive to modality and timing; the other theories in the literature are not. So only vicarious deployment currently predicts that the Apperly and Robinson phenomena should vary with the modality and timing of the agent’s information. Observed data match the predictions of the vicarious deployment theory (Fig. 2, top right panel). The other theories can be eliminated unless they can build in the required sensitivity to timing and modality.

To close the general discussion, we’d like to briefly relate our mental files theory to current discussions of the mental representation of beliefs and its development. One issue concerns when children become able to represent beliefs. Our default assumption was that this ability emerges around 4 years when children can provide correct answers to the test question in the standard false belief task. However, use of indirect measures like anticipatory looking (Clements & Perner, 1994; Southgate, Senju, & Csibra, 2007) or longer looking at unexpected outcomes (Onishi & Baillargeon, 2005) indicate a much earlier sensitivity to others’ belief around 14–18 months or even earlier. The cognitive basis of these early signs remains hotly disputed (Ruffman, 2014; Setoh, Scott, & Baillargeon, 2016; Wellman, 2014) and has recently been compounded by replication difficulties (Kulke & Rakoczy, 2017). One way of incorporating the distinction between early and later belief evidence into mental files theory is discussed by Perner (2016): Before children pass the standard test they may have vicarious files that are not vertically linked to their regular files. So they can show sensitivity to other’s beliefs when circumstances favour using these vicarious files (e.g., being absorbed in story events). But they lose sensitivity to others’ beliefs, for example, when an experimenter asks a question that disengages them from the agent’s perspective. When disengaged they use their own regular files. With the ability to link files comes the ability to switch at will between the perspective afforded by the regular files and the perspective afforded by the vicarious files. The ability to link files comes online around age 4, which underwrites success on explicit false belief tasks around that age.

Another recent development relevant to mental files theory is the mental files theory offered in Kovács (2016). While ours is a theory of *object* files – files that record what individuals know about specific objects – Kovács has offered a theory of *belief* files – files that track the contents of others’ beliefs. On Kovács’ theory a belief file is a structure with separate slots for the content of the belief and the agent who holds the belief (p. 525). Belief files enable their users to quickly and efficiently track the mental states of several agents in a rapidly changing world. “In the event that only the content of the belief has to be updated, one will replace the content component, and will not have to re-initiate the whole belief computation process” (p. 515). Belief files, as elaborated by Kovács so far, do not provide the distinction between representation of an object (the file) and what is known about the object (information on the file). This distinction is essential for us in order to make sense of (+ −) children’s curious incoherence in Apperley and Robinson’s Heinz scenario. In general, Kovács’ belief files seem designed to address our ability to quickly and automatically react to others’ mental states. In contrast, our approach pertains more to a reflective process whose products can be used in justifying people’s actions and answering questions by an experimenter.

## 5 Conclusion

Our results reveal important features of children’s mental file management. Mental files theory explains why children around 4 years of age pass the false belief test at the same time as they become able to process identity statements (Perner et al., 2011) and play the alternative naming game (Doherty & Perner, 1998; Perner & Leahy, 2016). It explains why mastery of the false belief task coincides with mastery of tasks that require understanding perspective, like level 2 visual perspective tasks (Flavell, Everett, Croft, & Flavell, 1981; Hamilton, Brindley, & Frith, 2009), the appearance-reality distinction (Courtin & Melot, 2005; Gopnik & Astington, 1988; Taylor & Carlson, 1997), and false direction signs (see Perner & Leekam, 2008, for review). Using only plausible assumptions about when children assign a vicarious file to an agent, we have shown that mental files theory also successfully explains why 4- to 6-year-olds give incoherent answers in Apperly and Robinson’s (1998) Heinz scenario and why that incoherence disappears given some very simple manipulations while elegantly linking improvement in the seen-after condition to the mastery of second-order problems.

## Footnotes

2 Friends of Fred Dretske (1969) may be reminded by this procedure of “non-epistemic seeing”. Heinz sees the eraser without knowing that it is an eraser, just like, Dretske’s example, Rosemary Woods saw the Watergate tape being erased without knowing – she claimed – that it was the Watergate tape that was being erased. It also captures the intuition behind “registration” (Apperly & Butterfill, 2009), “record of tracked events” (Perner, Rendl, & Garnham, 2007) or “experiential record” (Perner & Roessler, 2010) as proposals for children’s early sensitivity to belief.

3 Apperly and Robinson (1998) claim that the question, “Does Heinz know that the die is an eraser?” does not have both opaque and transparent interpretations, hence children do not struggle with this question (p. 296).

4 Rakoczy et al. (2015) also mention linguistic demands as another complication. Their comments apply to intensionality tasks like those by Russell (1987) that explicitly ask children to make metalinguistic judgements. Since our task did not require explicit metalinguistic judgements, we set this issue aside.

5 The (− −) children performed well on the memory question (93% correct) but struggled with the know questions (63% correct), while the (+ −) and the (+ +) children answered 97% of the memory and 97% of the know questions correctly, respectively. Problems with the know questions were most prominent in the youngest (− −) children (between 35 months and 44 months; n = 10) who responded correctly to only 24% of the know questions but gave correct answers to 83% of the memory questions, while older (− −) children (45 months and older; n = 21) had few problems, 82% and 98% correct answers, respectively. This is in line with past evidence that children answer knowledge questions correctly somewhat before they understand false belief (Hogrefe et al., Wimmer & Perner, 1986; Sodian et al., 2006).
